# Supplementary material for: Difference in Levels of Vitamin D between Indoor and Outdoor Athletes: A Systematic Review and Meta-Analysis
Source: Int J Mol Sci. 2023 Apr 20;24(8):7584. doi: 10.3390/ijms24087584 (PMC10147028; doi:10.3390/ijms24087584)
Supplement: Supplementary file 1 [file ijms-24-07584-s001.zip › ijms-2293124-supplementary.pdf]

Table S1. Queries used for each database search

Database	Query
Pubmed	(indoor OR outdoor OR inside OR outside OR interior OR exterior OR indoors OR outdoors OR internal OR external) AND ("vitamin D" OR "vitamin D"[MeSH] OR "D vitamin" OR "vitamin D deficiency" OR "vitamin D deficiency"[MeSH] OR "vitamin D insufficiency" OR "D hipovitaminosis" OR "hipovitaminosis D" OR "D hypovitaminosis" OR "hypovitaminosis D" OR "calcitriol" OR "calcitriol"[MeSH] OR "calcitriols" OR "calciferol" OR "calciferols" OR "cholecalciferol" OR "cholecalciferol"[MeSH] OR "cholecalciferols" OR "colecalciferol" OR "colecalciferols" OR "ergocalciferol" OR "ergocalciferol"[MeSH] OR "ergocalciferols" OR "ergocalciferols"[MeSH] OR calcidiol OR calcidiols OR calcifediol OR calcifediols OR calcifediol[MeSH]) AND (athlete OR athletes OR athletes[MeSH] OR sport OR sports OR sports[MeSH])
Embase	('indoor'/syn OR indoor OR 'outdoor'/syn OR outdoor OR inside OR outside OR interior OR exterior OR indoors OR outdoors OR internal OR external) AND ('vitamin d'/syn OR 'vitamin d' OR (('vitamin'/syn OR vitamin) AND d) OR 'd vitamin' OR (d AND ('vitamin'/syn OR vitamin)) OR 'hipovitaminosis d'/syn OR 'd hipovitaminosis'/syn OR 'hypovitaminosis d'/syn OR 'd hypovitaminosis'/syn OR 'calcitriol'/syn OR calcitriol OR calcitriols OR 'calciferol'/syn OR calciferol OR calciferols OR 'colecalciferol'/syn OR colecalciferol OR 'cholecalciferol'/syn OR cholecalciferol OR colecalciferols OR 'cholecalciferols'/syn OR cholecalciferols OR 'ergocalciferol'/syn OR ergocalciferol OR 'ergocalciferols'/syn OR ergocalciferols OR ‘calcidiol’/syn OR calcidiols OR calcifediol OR calcifediols) AND (‘athlete’/syn OR ‘athletes’/syn OR ‘sport’/syn OR ‘sports’/syn) AND [2012-2023]/py
Scopus	TITLE-ABS-KEY((indoor OR outdoor OR inside OR outside OR interior OR exterior OR indoors OR outdoors OR internal OR external) AND (vitamin d OR d vitamin OR "vitamin d" OR "d vitamin" OR vitamin D deficiency OR vitamin d insufficiency OR "hipovitaminosis d" OR "d hipovitaminosis" OR "hypovitaminosis d" OR "d hypovitaminosis" OR calcitriol OR calcitriols OR calciferol OR calciferols OR colecalciferol OR cholecalciferol OR colecalciferols OR cholecalciferols OR ergocalciferol OR ergocalciferols OR calcidiol OR calcidiols OR calcifediol OR calcifediols) AND (athlete OR athletes OR sport OR sports))
Science Direct	Field used: Title – abstract – key; (indoor OR outdoor OR inside OR outside OR interior OR exterior) AND ("vitamin d" OR calciferol) AND athlete
Proquest	(ABSTRACT(indoor) OR ABSTRACT(outdoor) OR ABSTRACT(inside) OR ABSTRACT(outside) OR ABSTRACT(interior) OR ABSTRACT(exterior) OR ABSTRACT(indoors) OR ABSTRACT(outdoors) OR ABSTRACT(internal) OR ABSTRACT(external)) AND (ABSTRACT("vitamin d") OR ABSTRACT("d vitamin") OR ABSTRACT("vitamin d deficiency") OR ABSTRACT("vitamin d insufficiency") OR ABSTRACT("hipovitaminosis d") OR ABSTRACT("d hipovitaminosis") OR ABSTRACT("hypovitaminosis d") OR ABSTRACT("d hypovitaminosis") OR ABSTRACT(calcitriol) OR ABSTRACT(calciferol) OR ABSTRACT(cholecalciferol) OR ABSTRACT(cholecalciferols) OR ABSTRACT(colecalciferol) OR ABSTRACT(colecalciferols) OR ABSTRACT(ergocalciferol) OR ABSTRACT(ergocalciferols) OR ABSTRACT(calcidiol) OR ABSTRACT(calcidiols) OR ABSTRACT(calcifediol)

	OR ABSTRACT(calcifediols)) AND (ABSTRACT(athlete) OR ABSTRACT(athletes) OR ABSTRACT(sport) OR ABSTRACT(sports))
Lilacs	(indoor OR outdoor OR inside OR outside OR interior OR exterior OR indoors OR outdoors OR internal OR external) AND ("vitamin d" OR "d vitamin" OR "vitamin d deficiency" OR "vitamin d insufficiency" OR "hipovitaminosis d" OR "d hipovitaminosis"OR "hypovitaminosis d" OR "d hypovitaminosis" OR calcitriol OR calciferol OR cholecalciferol OR cholecalciferols OR colecalciferol OR colecalciferols OR ergocalciferol OR ergocalciferols OR calcidiol OR calcidiols OR calcifediol OR calcifediols) AND (athlete OR athletes OR sport OR sports)
Cochrane Central Register of Controlled Trials	(indoor OR outdoor OR inside OR outside OR interior OR exterior OR indoors OR outdoors OR internal OR external) AND ("vitamin d" OR "d vitamin" OR "vitamin d deficiency" OR "vitamin d insufficiency" OR "hipovitaminosis d" OR "d hipovitaminosis" OR "hypovitaminosis d" OR "d hypovitaminosis" OR calcitriol OR calciferol OR cholecalciferol OR cholecalciferols OR colecalciferol OR colecalciferols OR ergocalciferol OR ergocalciferols OR calcidiol OR calcidiols OR calcifediol OR calcifediols) AND (athlete OR athletes OR sport OR sports)
ClinicalTrials.gov	Condition/disease: vitamin D<br>Other terms: athlete sport
International Clinical Trials Registry Platform (WHO)	vitamin d athlete
Katalog der Deutschen Nationalbibliothek	“Vitamin D”, “Cholecaliferol”, “Colecalciferol”, “Calcidiol”, “Calcitriol”,“Sportler”, “SportlerInnen”, “Athlet”, “Athleten” “AthletInnen”
EBSCO Open Dissertations	vitamin d athlete

Table S2. Newcastle-Ottawa risk of bias assessment for included cross-sectional studies.

Study	Sample representativeness	Sample size	Non-respondents	Ascertainment of exposure	Comparability (0/1/2 factors)	Assessment of outcome	Statistical test	Total (of 10)	Reference
Aydin2019	1	0	0	2	2	1	1	7	[40]
Bauer2018	0	0	0	2	2	0	1	5	[41]
Bauer2020	0	0	0	2	2	0	1	5	[42]
Fields2020	0	0	0	2	2	0	1	5	[43]
Geiker2017	0	0	0	2	1	0	1	4	[44]
Huang2021	1	0	0	2	1	0	1	5	[45]
Jakse2019	0	0	0	2	1	0	1	4	[46]
Kawashima2021	0	0	0	2	2	0	1	5	[47]
Kim2019	0	0	0	2	1	0	1	4	[48]
Ksiazek2018	0	0	0	2	0	0	1	3	[49]
Ksiazek2021	0	0	0	2	0	0	1	3	[50]
McGill2014	0	0	0	1	1	2	1	5	[51]
Mehran2016	1	0	1	1	1	2	1	7	[52]
Most2021	0	0	0	2	2	0	1	5	[53]
Peeling2012	0	0	0	2	2	1	1	6	[54]
Radovanovic2022	0	1	0	2	1	0	1	5	[55]
Ricart2021	0	0	0	2	2	0	1	5	[56]
Sariakcali2020	0	0	0	2	1	0	0	3	[57]
Sghaier2015	0	0	0	2	2	0	1	5	[58]
Valtuena2014	0	0	0	2	0	0	1	3	[59]
Wentz2016	0	1	0	2	2	0	1	6	[60]

Table S3. Newcastle-Ottawa risk of bias assessment for included cohort studies.

Study	Representative-ness	Selection of non-exposed cohort	Ascertainment of exposure	Outcome not present at start	Comparability (0/1/2 factors)	Assessment of outcome	Follow-up long enough	Adequacy of follow-up	Total (of 9)	Reference
Caroli2014	0	1	1	1	0	0	1	1	5	[61]
Fields2019	0	1	1	1	1	0	1	1	6	[62]
Galan2012	0	1	1	1	2	0	1	1	7	[63]
Haslacher 2016	1	1	1	1	2	0	1	1	8	[64]
Huggins2019	0	1	1	1	1	0	1	0	5	[65]
Krzywanski2016	0	1	1	0	0	0	1	1	4	[66]
Maruyama2016	0	1	1	1	2	0	1	1	7	[67]
Millward2020	1	1	1	1	2	1	1	1	9	[68]
Wilson2020	0	1	1	1	1	0	1	1	6	[69]
Valtuena2021	0	1	1	1	2	1	1	1	8	[70]

Table S4. Newcastle-Ottawa risk of bias assessment for included randomized controlled trial.

Study	Case definition adequate	Sample representativeness	Selection of controls	Definition of controls	Comparability (0/1/2)	Ascertainment of exposure	Same ascertainment of exposure for cases and controls	Non-response rate	Total (of 9)	Reference
Wyon 2015	1	1	1	0	2	0	1	0	6	[71]

Table S5. NHLBI risk of bias assessment for the included before-and-after study.

Study	Objective clearly stated	Selection criteria prespecified and clear	Representativeness	All eligible participants enrolled	Sample size	Test clearly described and consistent	Outcome measures prespecified, clear and consistent	Assessors blinded	Lost to follow-up <20%	Statistical tests	Interrupted time series	Individual-level data used	Total (of 12)	Reference
Valenti2022	1	1	1	0	0	1	1	0	1	1	0	0	7	[72]

Table S6. Results of Egger's test for funnel plot asymmetry in the complete set of results and in subgroups.

Subgroup	Intercept	Standard error	t statistic (df)	p-value
All	0.92	2.27	0.41 (91)	0.686
Indoor	0.96	1.36	0.7 (51)	0.485
Outdoor	2.09	4.28	0.49 (38)	0.628
Spring	-1.59	2.04	-0.78 (14)	0.449
Summer	0.028	7.98	0.00 (12)	0.997
Autumn	5.03	4.165	1.21 (18)	0.243
Winter	-1.40	2.25	-0.62 (20)	0.541

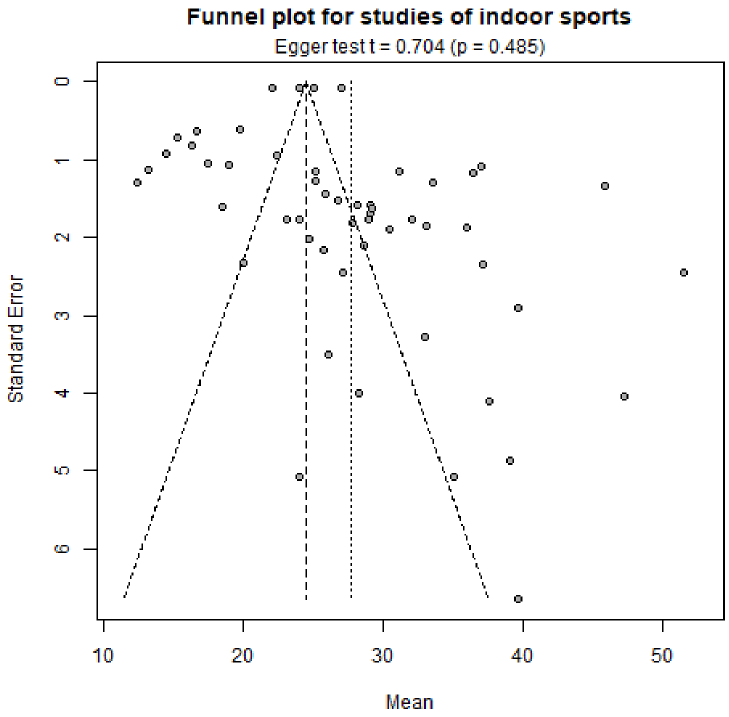

Figure S1. Funnel plot for all studies of indoor athletes. The distribution, while not funnel-shaped, is relatively symmetrical, which is supported by a non-significant Egger's test result

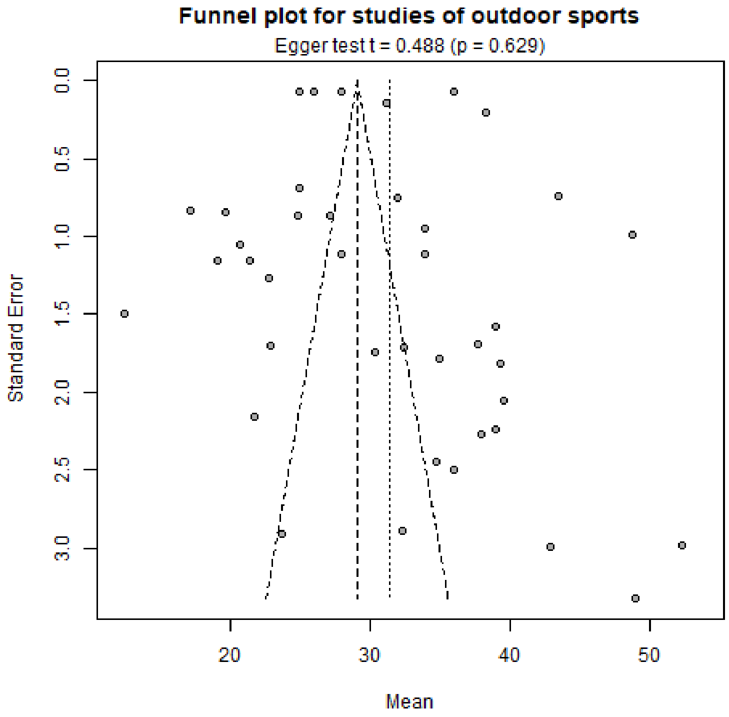

Figure S2. Funnel plot for all studies of indoor athletes. The distribution, while not funnel-shaped, is relatively symmetrical, which is supported by a non-significant Egger's test result

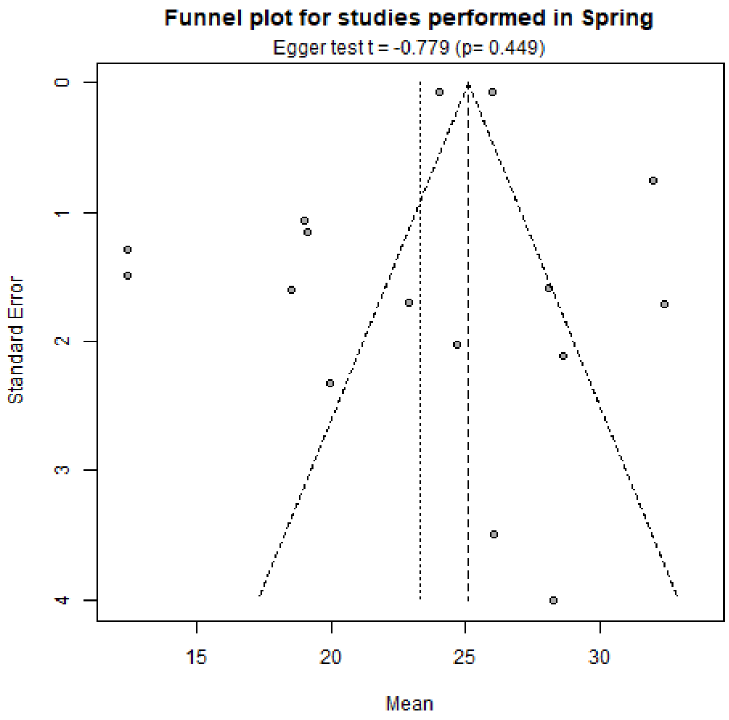

Figure S3. Funnel plot for all studies performed in spring. The distribution shows a slight left-asymmetry, but the result of Egger's test is not statistically significant

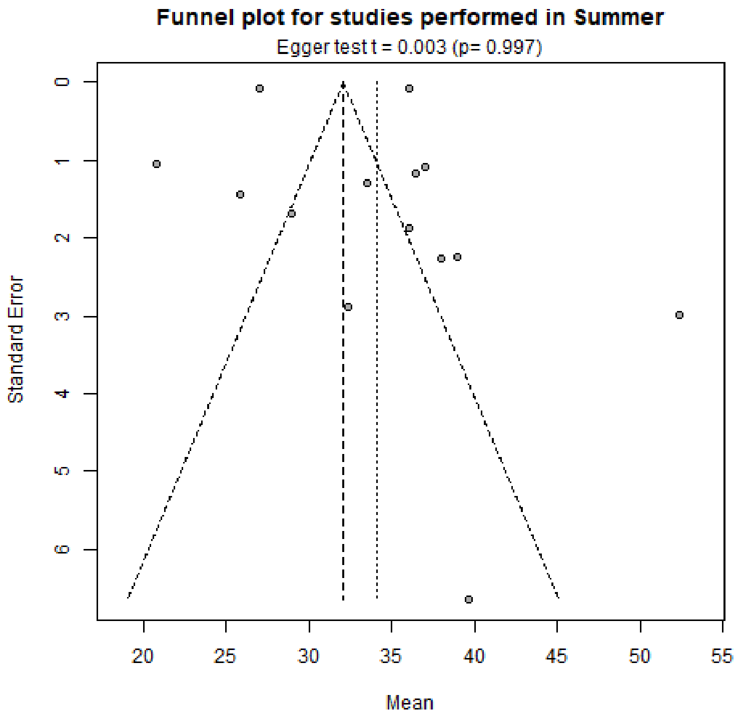

Figure S4. Funnel plot for all studies performed in summer. The distribution is relatively symmetrical, with a non-significant Egger's test result

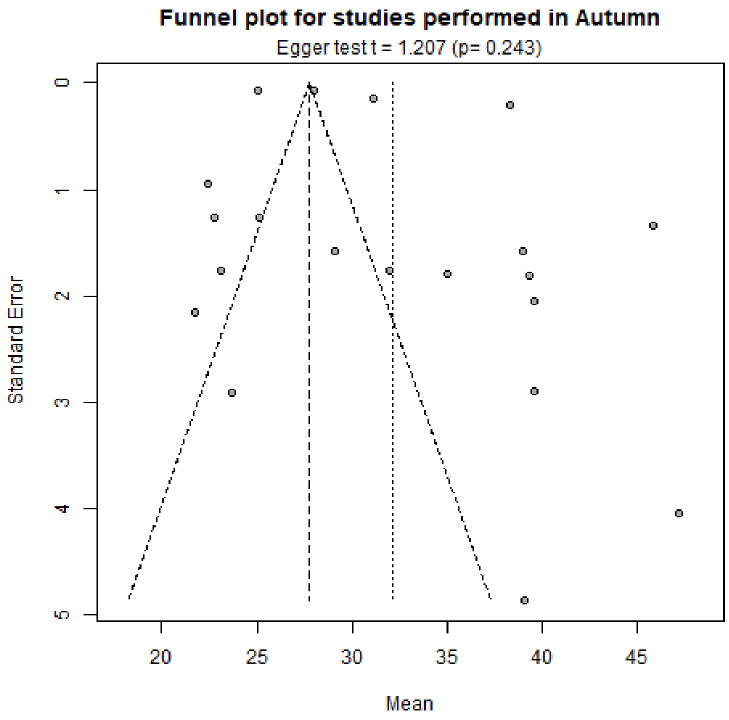

Figure S5. Funnel plot for all studies performed in autumn. The distribution is relatively right-asymmetrical, but Egger's test is not significant, even at the 0.1 level of confidence

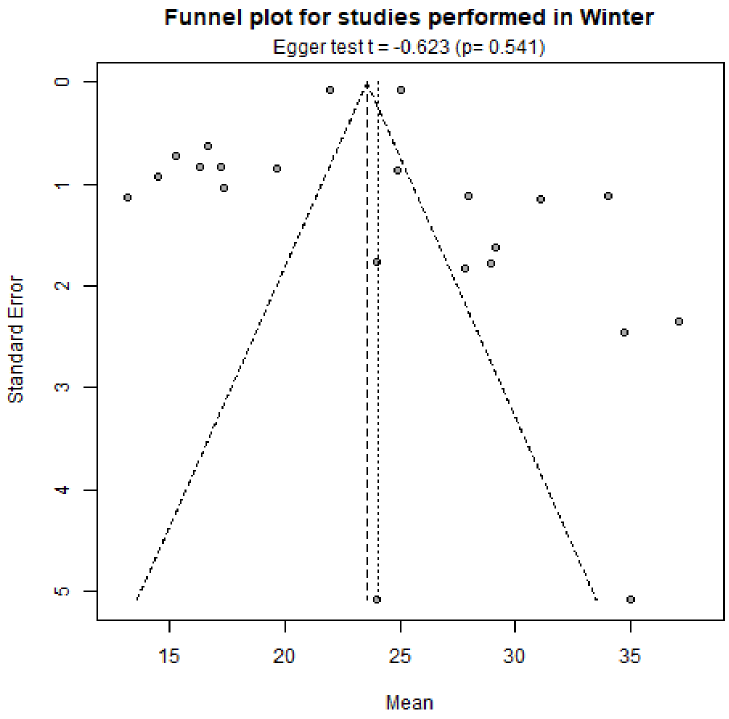

Figure S6. Funnel plot for all studies performed in winter. The distribution is slightly left-asymmetrical, but Egger's test is not significant, even at the 0.1 level of confidence

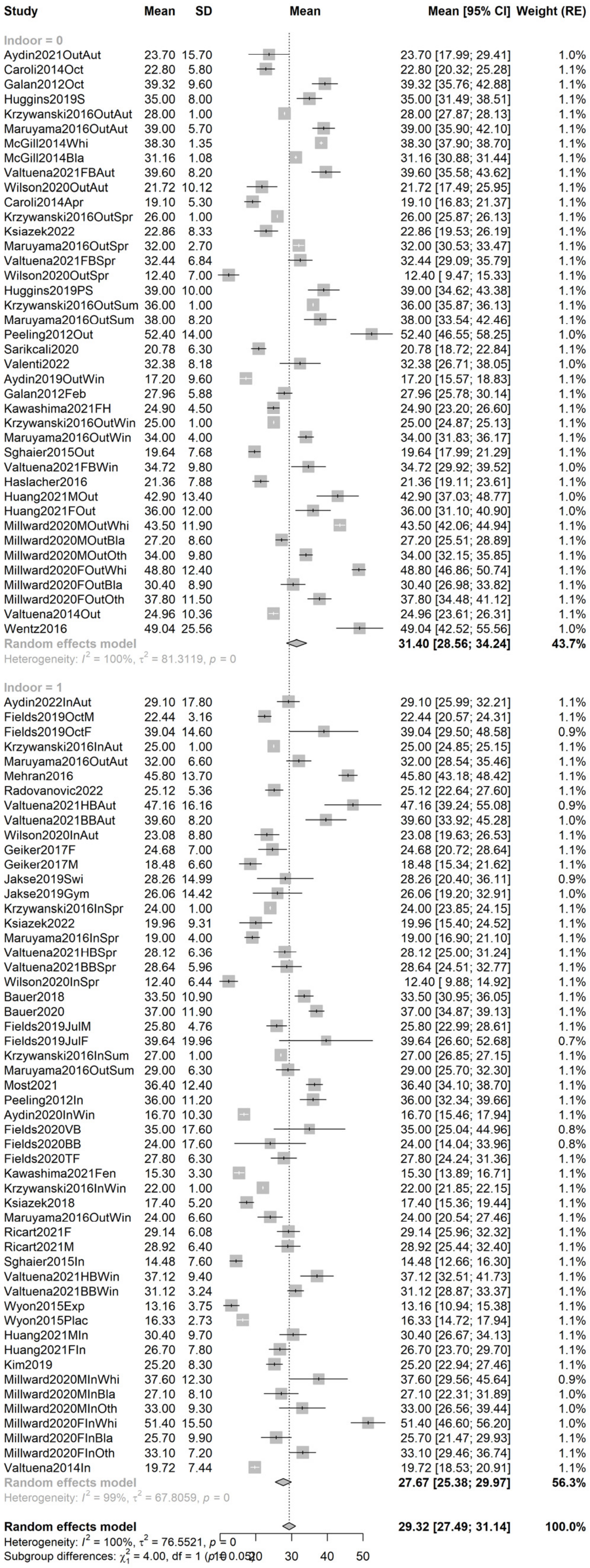

Figure S7. Forest plot of all included studies, grouped by type of sport (indoor/outdoor). Subgroup differences are only significant at a 0.1 level of confidence (test statistic = 4.00, df = 1, p = 0.052)
